# Supplementary material for: Field evaluation of a novel, rapid diagnostic assay, and molecular epidemiology of enterotoxigenic E. coli among Zambian children presenting with diarrhea
Source: PLoS Negl Trop Dis. 2022 Aug 5;16(8):e0010207. doi: 10.1371/journal.pntd.0010207 (PMC9385031; doi:10.1371/journal.pntd.0010207)
Supplement: S1 Fig — (DOCX) [file pntd.0010207.s001.docx]

# Supplementary Data

We observed a seasonal trend of ETEC over 12 months with high positivity rates between December to February (warm, rainy season) and a minor peak between April and May (dry season) (S1 Fig)

### Supplementary Figure 1. Seasonality of ETEC Infection Nov 2012 - Sep 2013 (S1 Fig)
